# Computational Prediction of Conformational B-Cell Epitopes from Antigen Primary Structures by Ensemble Learning

**DOI:** 10.1371/journal.pone.0043575

**Published:** 2012-08-21

**Authors:** Wen Zhang, Yanqing Niu, Yi Xiong, Meng Zhao, Rongwei Yu, Juan Liu

**Affiliations:** 1 School of Computer, Wuhan University, Wuhan, China; 2 School of Mathematics and Statistics, South-Central University for Nationalities, Wuhan, China; 3 Key Laboratory of Aerospace Information Security and Trust Computing, Ministry of Education, Wuhan, China; CSIR-Institute of Microbial Technology, India

## Abstract

**Motivation:**

The conformational B-cell epitopes are the specific sites on the antigens that have immune functions. The identification of conformational B-cell epitopes is of great importance to immunologists for facilitating the design of peptide-based vaccines. As an attempt to narrow the search for experimental validation, various computational models have been developed for the epitope prediction by using antigen structures. However, the application of these models is undermined by the limited number of available antigen structures. In contrast to the most of available structure-based methods, we here attempt to accurately predict conformational B-cell epitopes from antigen sequences.

**Methods:**

In this paper, we explore various sequence-derived features, which have been observed to be associated with the location of epitopes or ever used in the similar tasks. These features are evaluated and ranked by their discriminative performance on the benchmark datasets. From the perspective of information science, the combination of various features can usually lead to better results than the individual features. In order to build the robust model, we adopt the ensemble learning approach to incorporate various features, and develop the ensemble model to predict conformational epitopes from antigen sequences.

**Results:**

Evaluated by the leave-one-out cross validation, the proposed method gives out the mean AUC scores of 0.687 and 0.651 on two datasets respectively compiled from the bound structures and unbound structures. When compared with publicly available servers by using the independent dataset, our method yields better or comparable performance. The results demonstrate the proposed method is useful for the sequence-based conformational epitope prediction.

**Availability:**

The web server and datasets are freely available at http://bcell.whu.edu.cn.

## Introduction

Antigen-antibody interaction is a critical event in the immune process, and it can elucidate the underlying mechanism of immune recognition. The sites on antigens recognized and bound by B cell-produced antibodies are well known as B-cell epitopes [1]. The location of B-cell epitopes is useful for synthesizing peptides that can elicit the immune response with specific cross-reacting antibodies. For this reason, the identification of B-cell epitopes facilitates the design of the potentially safer peptide-based vaccines [2], [3]. B-cell epitopes can be classified into two categories: linear (continuous) epitopes and conformational (discontinuous) epitopes [4]. Linear epitopes are formed by continuous amino acid sequences, while conformational epitopes consist of residues that are distantly separated in the sequences but spatially proximal.

Recently, with the development of information science, computational methods for epitope recognition become an alternative to the wet experimental techniques, in order to save time and reduce cost. The study on linear epitope prediction started in 1970s, and some methods were proposed by using amino acid propensities [5]–[11]. In the last few years, machine learning methods were introduced into the linear epitope prediction with high accuracy [12]–[17]. Although the majority of all epitopes (about 90%) are conformational, the study on them began fairly late.

In the prediction work, conformational epitopes are usually defined based on the antigen-antibody distance. Specifically, the distance between two residues is measured by the minimal Euclidean distance between the centers of any of their non-hydrogen atoms, and an antigen residue separated from any antibody residue by a distance less than 4Å is defined as an epitope residue. Actually, the conformational epitopes in the computing community are structural epitopes. The computational methods help immunologists to identify the promising candidate residues that can constitute the epitope for the real application. Therefore, the development of computational methods is aimed to narrow the search for experimental validation, instead of replacing the experiments.

CEP [18] is the pioneer method for prediction of conformational epitopes, which uses the residue solvent accessibility. DiscoTope [19] exploits the surface accessibility, spatial information and amino acid statistics information to identify epitopes. PEPITO [20] combines amino acid propensities and half sphere exposure values at multiple distances to make prediction. ElliPro [21] is constructed using Thornton’s propensities and residue clustering. In SEPPA [22], two concepts ‘unit patch of residue triangle’ and ‘clustering coefficient’ are introduced to describe the local spatial context and spatial compactness. EPITOPIA [23], [24] combines structural and physicochemical features, and then uses naive Bayes classifier to make prediction. EPCES [25] uses the consensus score of several structural and physicochemical terms. EPSVR [26] uses support vector machine and combines various features for prediction. EPMeta [26] is a meta method that combines the outputs from existing servers. Liu et al. [27] adopted the logistic regression to predict the conformational epitopes. Zhang et al. proposed a random forest-based method by dealing with the imbalanced dataset and combining various features [28].

Although some structure-based computational methods have been developed for the epitope prediction, the application of these methods is undermined by the limited number of available antigen structures, and the experimental techniques that determine structures are costly and time-consuming. Recently, instead of making predictions based on structures, Ansari [29] made the first attempt on sequence-based conformational epitope prediction, and developed a server named ‘CBTOPE’.

In the paper, we follow the work pioneered by Ansari [29], and focus on two aspects concerning the sequence-based prediction. One is to explore more potential sequence-derived features relevant to conformational epitopes. The other is to effectively use various features which may share redundant information. In order to address these issues, we evaluate several sequence-derived features, which are ever used in the epitope prediction or similar tasks. Second, we consider the ensemble learning technique that can incorporate useful features, and the weighted scoring approach is adopted to build the prediction model.

## Methods

### Dataset

To our knowledge, there are two benchmark datasets widely used in the recent studies [23], [24], [25], [26]. One is Rubinstein’s bound structure dataset [23], [24]; the other is Liang’s unbound structure dataset [25], [26]. We compile 83 antigen sequences and 48 antigen sequences (named as ‘bound sequence dataset’ and ‘unbound sequence dataset’) respectively from above structure datasets, and used them as the main dataset.

In order to fairly compare our proposed method with a previously developed sequence-based CBTOPE [29], the sequence dataset that constructs CBTOPE server (named ‘main dataset’ in [29]) is adopted as well.

Moreover, to fairly test different public servers, we adopt Liang’s independent dataset [26], which contains 19 antigen structures with annotated real epitopes. Antigen structures are used to test the structure-based servers; the corresponding sequences are used to test the sequence-based servers.

### Instance Generation

The overlapping residue segments are generated from the antigen sequences, by using a sliding window of the length *L*. For simplify, let *L* to be an odd integer. For a sequence with *N* residues, a total of *N−L+1* segments are extracted, and each segment is labeled as positive or negative according to the state of its central residue (epitope residue or non-epitope residue). Obviously, there are much more negative instances than positive instances, and the instances are seriously imbalanced.

**Figure 1:**
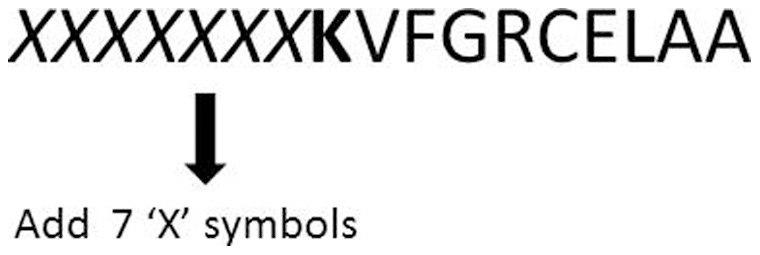
An example of adding ‘X’s at both terminals of a sequence. 7 ‘X’s are added at the left terminal of the given sequence (*L* = 15), the segment with the central residue ‘**K**’ is presented as ‘XXXXXXXKVFGRCEL’.

In order to deal with first 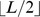 and last 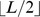
residues of the antigen sequences, 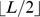 symbols ‘X’ are added at terminals of sequences. An example is shown by Fig. 1.

### Features

In order to apply machine learning techniques, the residue segments should be represented as feature vectors by using amino acid descriptors. In this paper, besides three groups of features (physicochemical propensities, sparse profile and amino acid composition) adopted in the CBTOPE [29], we evaluate more sequence-derived features. All features are described as follows.

Physicochemical propensities: lots of studies have suggested the close relationship between physicochemical propensities of amino acids and location of epitopes [5]–[11]. These physicochemical propensities are flexibility scale [5], hydrophilicity scale [6], surface exposed residue scale [7], polarity scale [8], beta-turn scale [9] and accessibility scale [10].

Sparse profile: sparse profile is a widely used representation of amino acids. Each amino acid type (20 common types in all) can be represented by a 20-bit binary string, in which the value at one bit is 1 and others are 0.

Amino acid composition: according to the previous study [23], some amino acid types are significantly overrepresented in epitopes, and others are underrepresented, thus the amino acid composition can be used to differentiate epitope regions from non-epitope regions. Here, we use the amino acid composition of the residue segments (also called as sliding windows or samples) extracted from the whole sequences. Ansari et al. [29] evaluated the feature in their sequence-based work, and proved its usefulness.

Amino acid function group: since contacts between antibodies and the antigens are mostly determined through functional moieties of the R-groups, functional moieties can influence the location of antibody-antigen binding sites [30], [31]. According to different R-groups, 20 amino acid types are classified into 13 classes (class 1: R, K; class 2: E, D; class 3: S, T; class 4: L, V, I; class 5: Q; N; class 6: W, F; class 7: A; class 8: C; class 9: G; class 10: H; class 11: M; class 12: P; class 13: Y). In order to take Ag-Ab interaction into consideration, we present a novel feature named ‘amino acid function group’, and use 13-bit binary strings to represent 13 functional classes.

Amino acid functional composition: by incorporating both amino acid function group and amino acid composition, we present a novel feature ‘amino acid functional composition’, which represents the percentage of each amino acid functional type in a sequence.

Evolutionary profile: Rubinstein studied the evolutionary conservation of epitopes [32], and revealed that epitopes are significantly less evolutionarily conserved than non-epitope regions. Therefore, the evolutionary conservation can help to differentiate epitopes from non-epitope regions. Here, the evolutionary conservation is represented by the position-specific scoring matrix (PSSM), which is obtained by aligning the target sequence against NCBI non-redundant reference sequences with PSI-BLAST tool. For an amino acid sequence with *L* residues, the PSSM has *L* rows and 20 columns. PSSM values in each row are rescaled to [0, 1] by the standard logistic function:
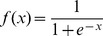



When using the evolutionary profile, a residue is represents by its corresponding 20-dimensional row vector in the matrix. This feature is widely used in the epitope prediction [23], [24], [25], [26] or similar tasks [33], [34], [35], [36] (protein-DNA binding prediction and protein-protein binding prediction).

Amino acid pair profile: The amino acid pair profile is usually observed to be associated with the protein functions [15], [23]. Amino acid pair profile of a sequence represents the percentage of each amino acid pair type.

Although structural information cannot be directly obtained from antigen sequences, some state-of-the-art tools can help to predict it. Here, the SABLE program [37] is adopted, for the online server and the standalone tool are publicly available [38]. With the given sequences as input, the software can predict the secondary structures and relative accessible surface areas (RASA) of residues. The predicted SS of a residue is denoted as H, E or C (helix, sheet, coil), and (1, 0, 0), (0, 1, 0) and (0, 0, 1) are respectively used to represent three types. The predicted RASA of a residue is a real value between 0 and 100, representing the percentage of exposed area of the residue over its full area.

### Random Forest and Imbalanced Data

Random forest (RF) is a machine learning method developed by Leo Breiman and Adele Cutler [39], which can be used for both classification and regression. Typically, a random forest (RF) is made up of many decision trees, which are constructed in the following way: the sampling technique is adopted to generate multiple samples from the dataset, and trees are constructed on these samples by selecting split features from a small random subset of features. The average vote of all trees is reported as the random forest prediction. RF has been widely used in the bioinformatics, and successfully solves lots of problems [40], [41], [42], [43]. Here, the random forest is used as the classification engine due to its efficiency and good generalization capability.

**Figure 2:**
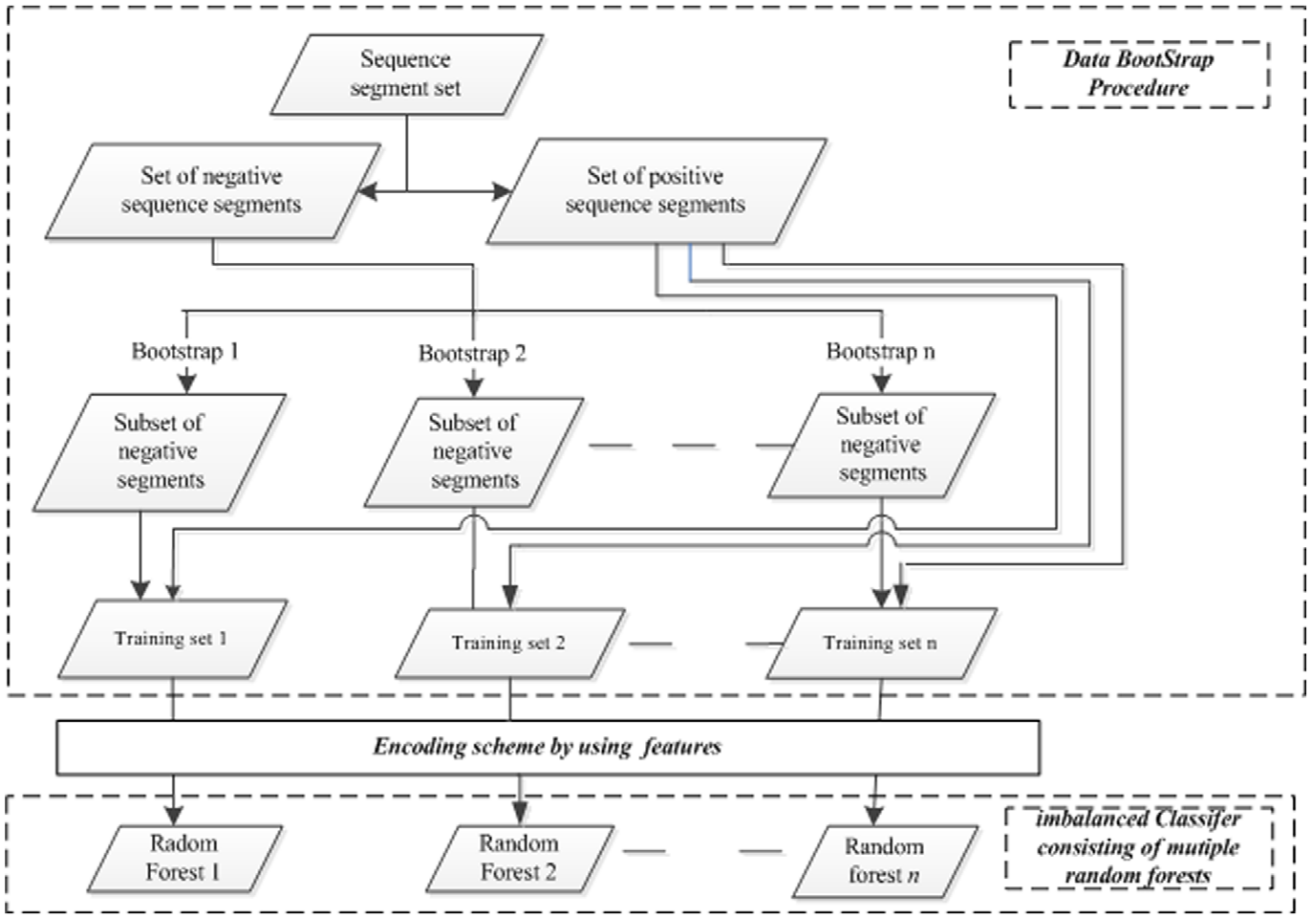
The model based on data bootstrap for the imbalanced dataset.

In fact, a great number of real datasets are imbalanced, in which the instances from one class take majority of the data. As shown in Fig. 2, a strategy based on the data bootstrap is used to deal with the imbalanced data. Thus, a model which consists of *n* random forests is constructed. When predicting an instance, votes yielded by *n* random forests are used as the predicted result. There is a parameter *n* which represents data sampling times, and it is set as the ratio of the number of positive instances divided by number of negative instances. The data bootstrap procedure and random forests are implemented by WEKA package [44], and default parameters are adopted.

### The Ensemble Model for Conformational Epitope Prediction

Ensemble learning is a useful technique that aggregates multiple machine learning models to achieve overall prediction accuracy as well as better generalization [45]. Recently, there is an increasing use of ensemble learning methods in the field of bioinformatics [46]–[49], because of their unique advantages in dealing with high-dimensional and complicated data. In this paper, we use the ensemble learning technique to exploit various features, and then develop the sequence-based prediction model.

**Figure 3:**
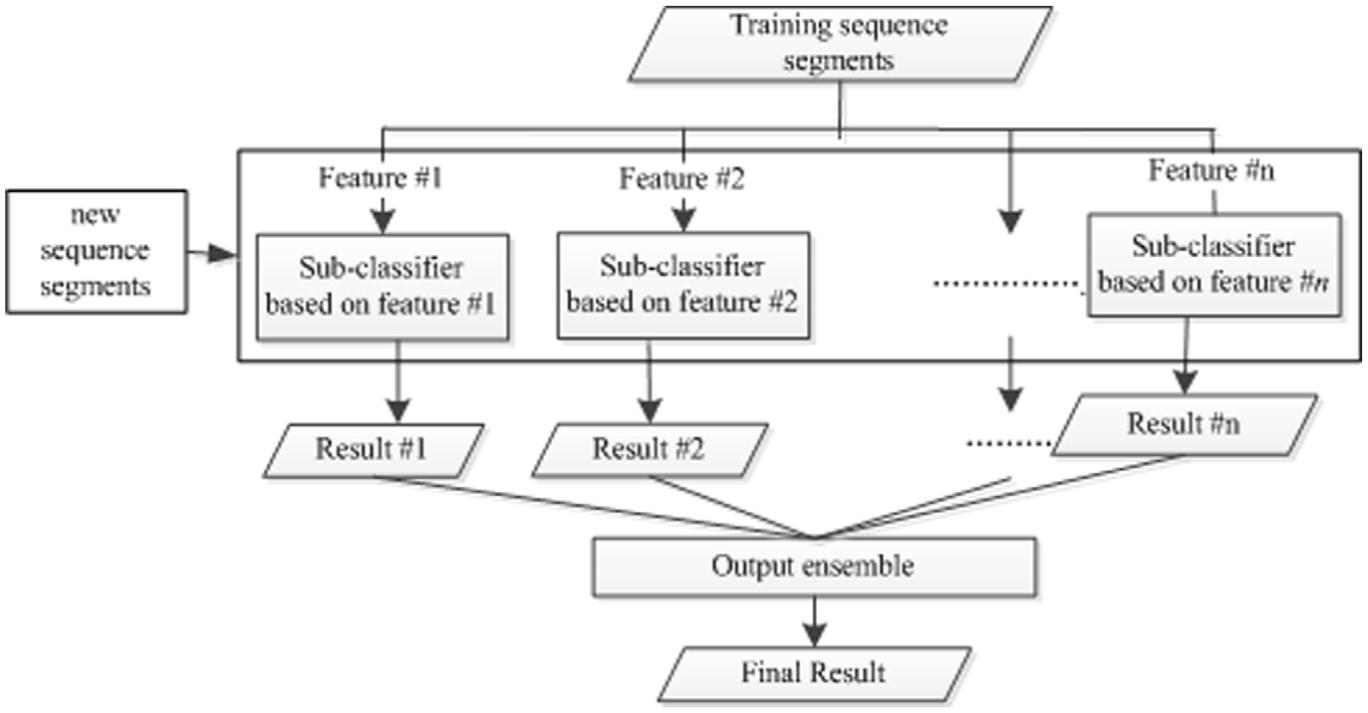
The general schematic diagram of the ensemble model.

Since a sequence segment can be encoded into different feature vectors by using different features, multiple classifiers can be constructed and used as the sub-classifiers for ensemble learning. In order to integrate various features, the ensemble model can be constructed by combining the outputs of different sub-classifiers. Fig. 3 shows the general flowchart of an ensemble model. Various strategies can be used to combine the sub-classifiers. Here, we adopt a simple strategy named weighted scoring, and the similar strategy is ever used in the protein-protein prediction [49]. The weighted scoring approach includes two steps: data normalization and score combination.

Given an instance, each sub-classifier will produce a score, and then these scores are normalized by the Z-score function, and transformed by *tanh* function [50].
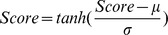
where μ and σ are the mean and the standard deviation of scores produced by the sub-classifiers.

Further, a weight is assigned to the normalized score yielded by a sub-classifier, and the sum of weighted scores is adopted as the final prediction.
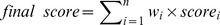



Where 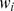 is the weight for the 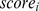 from sub-classifier #*i,* 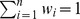 and 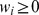.

In order to deal with the first 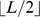
and last 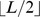 resides of an antigen sequence (the window length is *L*), the composition profile-based model is used.

### Performance Evaluation Metrics

The performance of the models is evaluated by the leave-one-out cross validation (LOOCV). With respect to our study, the LOOCV procedure is slightly different. Each time, the sequences from *n*-1 antigens are used to train the model, and the sequences from one antigen (an antigen may have multiple chains) are used to test the model.

The performance of models is measured by several metrics, i.e. sensitivity (SN), specificity (SP), accuracy (ACC), F-measure (F) and area under ROC curve (AUC). Here, AUC is used as the primary evaluation metric, for it can measure the general performance of models regardless of any threshold.
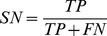


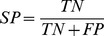


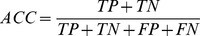


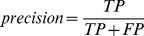


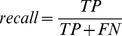


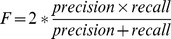
where 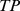, 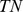, 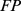 and 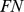 are the number of true positives, the number of true negatives, the number of false positives and the number of false negatives.

## Results and Discussion

In this section, we evaluate various features and identify the candidate features for the sequence-based prediction. Further, we investigate how to build the high-accuracy and reliable model based on these features.

### The Evaluation of Various Features

Before building prediction models, a fixed-length window is shifted over antigen sequences to generate overlapping segments as instances. Since the window length may influence the performance of models, the window lengths ranging from 5-residue to 15-residue are considered. Table 1 and table 2 demonstrate the prediction performance of individual feature-based models on the bound and the unbound sequence dataset.

**Table 1:** Performance of individual feature-based models for the bound sequence dataset, evaluated by LOOCV.

Window	#1	#2	#3	#4	#5	#6	#7	#8	#9	Average
5	0.604	0.580	0.601	0.601	0.673	0.599	0.583	0.617	0.637	0.611
7	0.617	0.575	0.607	0.609	0.678	0.600	0.578	0.613	0.640	0.613
9	0.632	0.576	0.598	0.609	0.678	0.613	0.589	0.607	0.650	0.617
11	0.622	0.565	0.593	0.597	0.673	0.593	0.564	0.606	0.648	0.607
13	0.633	0.576	0.603	0.599	0.672	0.602	0.5687	0.613	0.657	0.614
15	0.623	0.543	0.603	0.598	0.674	0.600	0.539	0.607	0.662	0.605

Physicochemical propensities (#1), amino acid composition (#2), amino acid function group (#3), amino acid functional composition (#4), evolutionary profile (#5), sparse profile (#6), amino acid pair profile (#7), secondary structure (#8), relative accessible surface area (#9).

**Table 2:** Performance of individual feature-based models for the unbound sequence dataset, evaluated by LOOCV.

Window	#1	#2	#3	#4	#5	#6	#7	#8	#9	Average
5	0.572	0.522	0.572	0.575	0.639	0.571	0.546	0.600	0.617	0.579
7	0.592	0.544	0.585	0.592	0.632	0.575	0.522	0.609	0.624	0.586
9	0.603	0.543	0.581	0.585	0.635	0.575	0.531	0.616	0.627	0.588
11	0.606	0.556	0.601	0.597	0.633	0.579	0.541	0.611	0.626	0.595
13	0.606	0.558	0.584	0.583	0.625	0.572	0.543	0.595	0.621	0.588
15	0.604	0.520	0.584	0.581	0.626	0.577	0.554	0.586	0.626	0.584

Physicochemical propensities (#1), amino acid composition (#2), amino acid function group (#3), amino acid functional composition (#4), evolutionary profile (#5), sparse profile (#6), amino acid pair profile (#7), secondary structure (#8), relative accessible surface area (#9).

Although the performance of individual feature-based models varies over the increasing window length, an overall tendency can be observed. Generally speaking, the performance will go up as the window length increases until reaching a peak, and then it will decrease. However, there is no consistent optimal window length (reaching peak performance) for all features. For the bound sequence dataset, the average performance of all individual feature-based models reaches peak when using the 9-residue window. For the unbound sequence dataset, the average performance of models with the 9-redisue window is close to the best (yielded by the 11-resuidue window). For simplicity, the 9-residue window is adopted in the following study.

**Figure 4:**
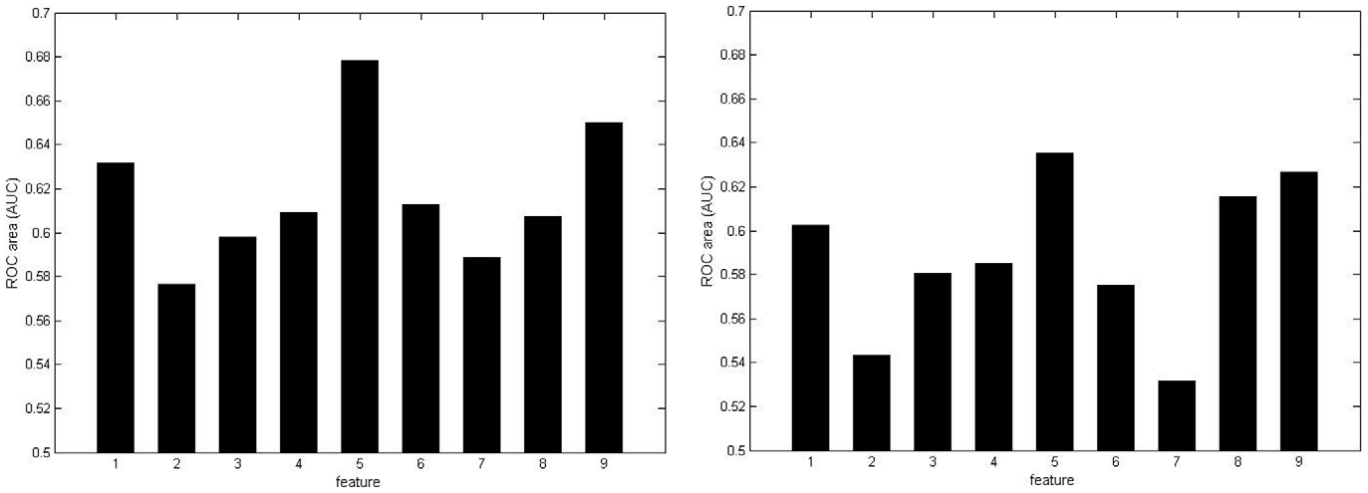
The feature rank evaluated by LOOCV (left: bound sequence dataset, right: unbound sequence dataset). Physicochemical propensities (#1), amino acid composition (#2), amino acid function group (#3), amino acid functional composition (#4), evolutionary profile (#5), sparse profile (#6), amino acid pair profile (#7), secondary structure (#7), relative accessible surface area (#8).

As shown in Fig. 4, various features can be ranked by the performance of individual feature-based models. For the bound sequence dataset, the evolutionary profile, predicted relative accessible surface area and physicochemical propensities produce better results than other features. The features can be listed in the descending order of their performance as evolutionary profile, predicted relative accessible surface area, physicochemical propensities, sparse profile, function composition, predicted secondary structure, amino acid pair profile. The similar conclusion can be drawn for the unbound sequence dataset.

In the sequence-based prediction, it is necessary to study the sequence-predicted structural values (by Sable [38]) and evaluate their effect. The RASA and SS calculated from crystal structures by DSSP software [51] can be approximately taken as the real structural value. We use real structural values and sequence-predicted structural values to build the prediction models, and make comparison. As expected, the real RASA produces better results than the sequence-predicted RASA (0.688 versus 0.650 on the bound dataset). However, the sequence-predicted SS yields better results than the real SS (0.608 versus 0.509). The results suggest the sequence-based prediction can reduce the influence of conformational change in some degree.

The study in the section indicates all features have the ability of differentiating epitope regions from non-epitope regions. Since the amino acid functional composition incorporates both amino acid composition and amino acid group, seven groups of features including physicochemical propensities, evolutionary profile, amino acid functional composition, sparse profile, amino acid pair, sequence-predicted secondary structure and sequence-predicted relative solvent accessibility are used as candidates for the development of prediction models.

### The Study on the Direct Feature Combination

From the perspective of information science, the combination of various features can lead to better results than the individual features. Emerging various feature vectors is an popular way of the direct feature combination, and its usefulness is proved by many applications in bioinformatics [25]–[28] [33]–[36].

However, as shown in table 3, the direct combination of the high-ranked features cannot produce better results than the best individual feature-based models for the bound sequence dataset, and the performance instead decreases. According to the Table 4, some feature combinations make improvement for the unbound sequence dataset, but more features cannot necessarily contribute to better performance. As a result, merging feature vectors can not effectively utilize various features for the sequence-based epitope prediction, because of the redundant and even conflicting information between these features. Therefore, we seek for another feasible approach to exploit all candidate features.

**Table 3:** Performance of models based on direct feature combination for the bound sequence dataset, evaluated by LOOCV.

Feature	F	SN	SP	ACC	AUC
A	0.311	0.671	0.675	0.680	0.678
A+B	0.313	0.670	0.685	0.689	0.676
A+B+C	0.311	0.662	0.678	0.685	0.680
A+B+C+D	0.309	0.608	0.723	0.719	0.680
A+B+C+D+E	0.312	0.650	0.695	0.698	0.676
A+B+C+D+E+F	0.309	0.637	0.713	0.711	0.677
A+B+C+D+E+F+G	0.307	0.681	0.658	0.669	0.669

A: evolutionary profiles; B: predicted relative accessible surface area; C: physicochemical propensities; D: sparse profile; E: function composition; F: predicted secondary structure; G: amino acid pair.

**Table 4:** Performance of models based on direct feature combination for the unbound sequence dataset, evaluated by LOOCV.

Feature	F	SN	SP	ACC	AUC
A	0.292	0.636	0.643	0.654	0.635
A+B	0.288	0.628	0.653	0.658	0.634
A+B+C	0.283	0.644	0.636	0.646	0.633
A+B+C+D	0.294	0.661	0.637	0.647	0.648
A+B+C+D+E	0.293	0.630	0.653	0.663	0.641
A+B+C+D+E+F	0.289	0.646	0.623	0.638	0.642
A+B+C+D+E+F+G	0.297	0.607	0.669	0.678	0.646

A: Evolutionary profiles; B: predicted relative accessible surface area; C: predicted secondary structure; C: physicochemical propensities; D: amino acid function composition; F: Sparse profile; G: Amino acid pair.

### The Performance of Ensemble Learning-based Models

In order to combine various features, we adopt the ensemble learning technique (described in the ‘Methods’ section) to build the prediction models. Individual feature-based models are used as the sub-classifiers, and the weighted sum of outputs given by sub-classifiers is used as the prediction.

In the paper, the weights assigned to different sub-classifiers can be determined by the grid search, in which the sum of weights is 1 and step size of weights is 0.05. For the time efficiency, the optimal weights are determined on the bound sequence dataset (the 9-residue window is adopted), and are further used for the unbound sequence dataset and other datasets.

**Figure 5:**
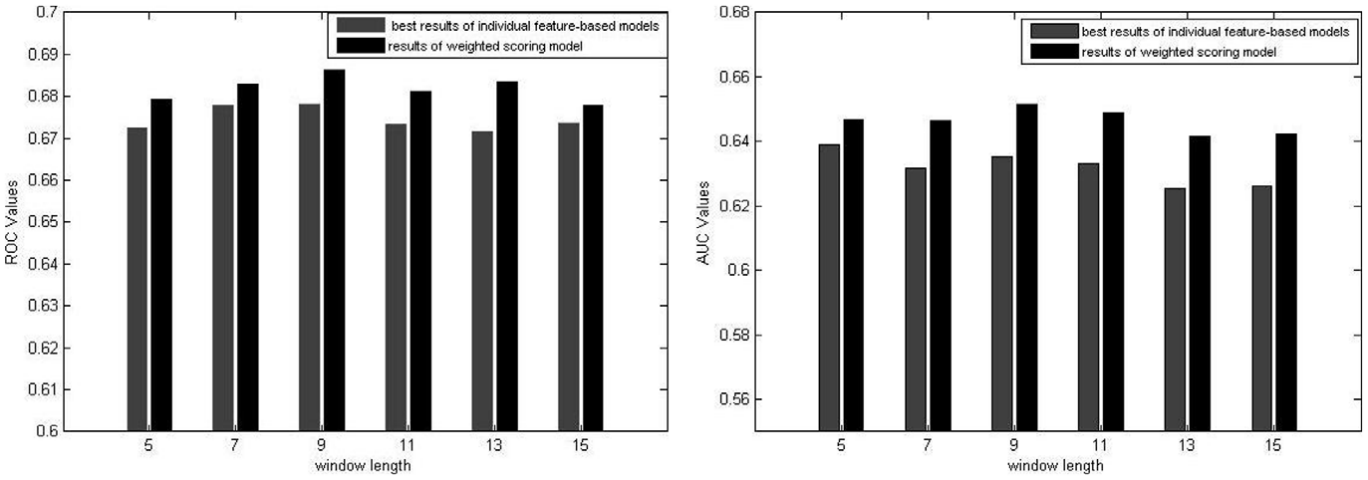
The LOOCV comparison between the ensemble model and the best individual feature-based models in terms of different window lengths. Optimal Weights for sub-classifier: 0.1 for physicochemical propensities, 0.0 for amino acid functional composition, 0.5 for evolutionary profiles, 0.0 for sparse profile, 0.1 for SS, 0.2 for RASA, 0.1 for amino acid pair profile. AUC scores of the ensemble model using the 9-residue window: 0.687 for bound dataset, 0.651 for unbound dataset.

As shown in Fig. 5, the ensemble model can produce consistently better results than the best individual feature-based models when using the windows of different lengths. Admittedly, the improvement is not significant and quite limited. However, due to the difficulty of epitope prediction, the reported accuracy of all existing methods is quite low. Therefore, we have to exploit useful features to achieve higher accuracy.

More importantly, the weighted scoring-based model has some advantages. First, the ensemble model provides a flexible frame that incorporates individual feature-based classifiers. For example, if we set 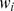 as 1 and others as 0, the ensemble model only uses the #i feature. Second, the ensemble model can select the features by itself and integrate them based on the discriminative power. According to the optimal weights, we can approximately know the components of the ensemble model. Therefore, this ensemble model is not only easy to implement but also easy to explain.

**Table 5:** The performance of different servers for the independent dataset.

Server Type	Data for server construction	Server	Available	Mean AUC
Structured-based	Bound structure	DiscoTope	http://www.cbs.dtu.dk/services/DiscoTope/	0.579
		SEPPA	http://lifecenter.sgst.cn/seppa/	0.589
		EPITOPIA	http://epitopia.tau.ac.il/	0.572
		BPredictor	http://code.google.com/p/my-project-bpredictor/	0.587
	Unbound structure	EPCES	http://sysbio.unl.edu/EPCES/	0.569
		EPSVR	http://sysbio.unl.edu/EPSVR/	0.606
Sequence-based	Sequence dataset	CBTOPE	http://www.imtech.res.in/raghava/cbtope/	0.607
	*Bound sequence*	*Our model^1^*		0.600
	*Unbound sequence*	*Our model^2^*		0.601
	CBTOPE *dataset*	*Our model^3^*		0.632

Our model^1^ is constructed on the sequence dataset compiled from the bound structures; our model^2^ is constructed on the sequence dataset compiled from the unbound structures; our model^3^ is constructed on the dataset, which was used for CBTOPE.

Besides the weighted scoring, other ensemble learning approaches such as mean scoring and median scoring are considered. According to our study, the weighted scoring approach yields best results among all ensemble approaches. The details of these approaches are provided in Table S1.

### Comparison with other Methods

To our knowledge, there are some conformational epitope prediction methods with publicly available web servers. These methods are CEP [18], DiscoTope [19], ElliPro [21], SEPPA [22], Epitopia [24], EPSVR [25], EPCES [26], EPMeta [26] and CBTOPE [29]. Except CBTOPE, all methods are trained on the structures and use the structures to make prediction. Here, we adopt the most recent methods DiscoTope, SEPPA, Epitopia, EPSVR, EPCES and CBTOPE as the benchmark methods for comparison.

As far as we know, some structure-based methods are trained and evaluated on the bound dataset (DiscoTope, SEPPA, Epitopia), the others are constructed and tested on the unbound dataset (EPSVR, EPCES). Therefore, we directly compare our method with the methods whose LOOCV results for these datasets are reported. On the same bound dataset and using exactly the same LOOCV assessment measures, DiscoTope and Epitopia produce the mean AUC scores of 0.60 and 0.59 (according to Rubinstein’s study [26]), and BPredictor [28](our previous method) yields the mean AUC score of 0.633. Here, the proposed sequence-based model produces the mean AUC score of 0.687. Additionally, we compare our model with the unbound structure-based methods. Evaluated by the same unbound dataset and evaluation measure, EPSVR [25], EPCES [26], and BPredictor [28] give out the LOOCV AUC scores of 0.670, 0.644, and 0.654, while the proposed sequence-based model yields the LOOCV AUC score of 0.651. Although EPSVR produces the best result, it is important to note that EPSVR adopts the best parameters of SVR for the LOOCV evaluation. Considering the fact that we use the default parameters of RF, our sequence-based method produces the comparable performance. Therefore, when compared with the structure-based methods in terms of LOOCV evaluation, our method produces better or comparable performance.

Currently, only one sequence-based method (CBTOPE) has been developed by Ansari to predict the conformational epitopes [29]. In CBTOPE, physicochemical propensities, sparse profile and amino acid composition are used to encode overlapping residue segments, thus support vector machine is adopted to construct prediction models. The amino acid composition-based model produces the best performance. In our study, we consider these features as well, and use them as the components of our ensemble model. The results in the Fig. 5 show the ensemble model yields better results than any individual feature-based model. However, the LOOCV scores of CBTOPE are not reported in [29]. Therefore, we can not directly compare our method with CBTOPE in terms of LOOCV evaluation. As an alternative, we try to compare our method with CBTOPE server in the following independent dataset testing.

In order to test real predictive power, our method and the benchmark servers are tested by an independent dataset, and results are shown in table 5. Here, we train our sequence-based models on the bound sequence dataset, the unbound sequence dataset and Ansari’s sequence dataset respectively, and then use them to predict the independent dataset. Three models produce the mean AUC scores of 0.60, 0.601, and 0.632. When compared with structure-based servers that are constructed on the bound and unbound datasets, our model can yield better or comparable performance. Here, we must emphasize, the sequence-based prediction is an alternative to the structure-based prediction in the absence of structures. Theoretically, the antigen structure can bring more information to build robust prediction models. However, the results suggest the sequence-based method can give out satisfying results by only using sequence information. Trained on the same dataset, our model gives out obviously better performance than the sequence-based CBTOPE (mean AUC score: 0.632 VS 0.607) for the independent dataset. Specifically, our model produces better results on 12 out of 19 antigen sequences (details shown in Table S2). Therefore, our ensemble model that incorporates various features produces more robust performance than the CBTOPE which only uses an individual feature.

According to the pairwise t-student test, the differences between our method and benchmark servers, as well as the differences between benchmark servers, are not statistically significant. The same results are reported in the previous study [26], [28]. As far as we know, the statistical analysis depends on the great number of samples. However, the limited number of available antigen-antibody complex structures is one of the main obstacles in the epitope prediction, thus leads to the result.

Generally speaking, the proposed sequence-based method produces comparable or better performance when compared with the structure-based methods, and makes improvement over the existing sequence-based method. More importantly, our method can predict the conformational epitopes from primary sequences in the absence of antigen structures, and has more practical values.

### Conclusions

Most conformational epitope prediction models are constructed on the antigen-antibody structures, and use antigen structures to make prediction. However, only a small number of antigen structures are available. Therefore, we attempt to predict conformational epitopes from antigen sequences. This paper systematically evaluates several sequence-derived features, and selects some features as candidates for modeling. In order to effectively combine candidate features, we develop an ensemble learning model based on the weighted scoring strategy. When compared with the existing sequence-based method and structure-based methods, our method demonstrates comparable or better performance. In conclusion, our method is a promising tool to predict the conformational epitopes from antigen sequences. The web server and datasets are freely available at http://bcell.whu.edu.cn.

## Supporting Information

Table S1 Performance of the models based on different ensemble learning strategies, evaluated by LOOCV.
(DOCX)

Table S2 The AUC scores produced by different servers for the independent dataset.
(DOCX)
