# Supplementary material for: Computational Prediction of Conformational B-Cell Epitopes from Antigen Primary Structures by Ensemble Learning
Source: PLoS One. 2012 Aug 21;7(8):e43575. doi: 10.1371/journal.pone.0043575 (PMC3424238; doi:10.1371/journal.pone.0043575)
Supplement: Table S1 — Performance of the models based on different ensemble learning strategies, evaluated by LOOCV. (DOCX) [file pone.0043575.s001.docx]

Table S1 Performance of the models based on different ensemble learning strategies, evaluated by LOOCV

|  | ensemble approach | F | SN | SP | ACC | AUC |
| --- | --- | --- | --- | --- | --- | --- |
| Bound sequence dataset | Mean scoring | 0.3194 | 0.6697 | 0.6795 | 0.6854 | 0.6745 |
|  | Median scoring | 0.3100 | 0.6475 | 0.6789 | 0.6856 | 0.6629 |
|  | Random Forest | 0.2880 | 0.6448 | 0.6220 | 0.6321 | 0.6241 |
|  | Logical regression | 0.1484 | 0.1840 | 0.9257 | 0.8569 | 0.5549 |
| Unbound sequence dataset | Mean scoring | 0.3051 | 0.6020 | 0.6957 | 0.6970 | 0.6454 |
|  | Median scoring | 0.2815 | 0.6235 | 0.6470 | 0.6563 | 0.6306 |
|  | Random Forest | 0.2787 | 0.5909 | 0.6415 | 0.6488 | 0.6138 |
|  | Logical regression | 0.1356 | 0.1652 | 0.8998 | 0.8201 | 0.5325 |

In addition to the weighted scoring, some ensemble learning approaches are considered. First of all, average scoring and median scoring are used. The average scoring approach uses the mean of scores yielded by sub-ensemble classifiers; the median scoring is the score in the median rank of all scores. Then, we consider the machine learning-based ensemble approach. Machine learning-based ensemble approach uses the scores from sub-classifiers and the real labels respectively as the inputs and outputs, thus the models exploring the relationship between predicted scores and the real labels are constructed by using machine learning methods. Here, random forest and logical regression are used to construct models. Logic regression is an adaptive regression methodology that attempts to construct predictors as Boolean combinations of binary covariates. The results of models using different ensemble learning strategies are shown in Table 1. Generally speaking, the weighted scoring approach yields best results among all ensemble approaches.
