# Supplementary material for: Computational Prediction of Conformational B-Cell Epitopes from Antigen Primary Structures by Ensemble Learning
Source: PLoS One. 2012 Aug 21;7(8):e43575. doi: 10.1371/journal.pone.0043575 (PMC3424238; doi:10.1371/journal.pone.0043575)
Supplement: Table S2 — The AUC scores produced by different servers for the independent dataset. (DOCX) [file pone.0043575.s002.docx]

**Table S2 The AUC scores produced by different servers for the independent dataset**

| Antigen | DiscoTope | EPITOPIA | EPCES | EPSVR | SEPPA | BPredictor | BETOPE | Our model^1^ | Our model^2^ | Our model^3^ |
| --- | --- | --- | --- | --- | --- | --- | --- | --- | --- | --- |
| 1eku | 0.490 | 0.612 | 0.635 | 0.489 | 0.794 | 0.662 | 0.447 | 0.626 | 0.441 | 0.434 |
| 1mbn | 0.541 | 0.777 | 0.589 | 0.609 | 0.830 | 0.769 | 0.457 | 0.455 | 0.462 | 0.529 |
| 1av1 | 0.874 | 0.653 | 0.457 | 0.876 | 0.610 | 0.666 | 0.537 | 0.532 | 0.500 | 0.620 |
| 1pv6 | 0.572 | 0.715 | 0.502 | 0.341 | 0.639 | 0.678 | 0.996 | 0.743 | 0.722 | 0.997 |
| 1al2 | 0.850 | 0.938 | 0.877 | 0.870 | 0.756 | 0.701 | 0.772 | 0.829 | 0.818 | 0.785 |
| 2gmf | 0.564 | 0.345 | 0.678 | 0.691 | 0.299 | 0.567 | 0.571 | 0.530 | 0.437 | 0.382 |
| 1a7c | 0.656 | 0.656 | 0.649 | 0.550 | 0.706 | 0.581 | 0.522 | 0.719 | 0.715 | 0.723 |
| 1y8o | 0.322 | 0.853 | 0.483 | 0.626 | 0.225 | 0.654 | 0.611 | 0.370 | 0.524 | 0.585 |
| 1og5 | 0.310 | 0.473 | 0.245 | 0.413 | 0.275 | 0.826 | 0.359 | 0.250 | 0.304 | 0.315 |
| 1jeq | 0.635 | NA | 0.419 | 0.679 | 0.808 | 0.245 | 0.529 | 0.523 | 0.756 | 0.445 |
| 1dab | 0.633 | 0.329 | 0.743 | 0.770 | 0.596 | 0.939 | 0.662 | 0.742 | 0.734 | 0.688 |
| 1w7b | 0.632 | 0.536 | 0.267 | 0.267 | 0.394 | 0.548 | 0.626 | 0.745 | 0.622 | 0.708 |
| 1ly2 | 0.597 | 0.458 | 0.534 | 0.617 | 0.585 | 0.755 | 0.543 | 0.693 | 0.664 | 0.618 |
| 1rec | 0.359 | 0.575 | 0.337 | 0.308 | 0.417 | 0.450 | 0.220 | 0.430 | 0.506 | 0.553 |
| 1nu6 | 0.526 | 0.814 | 0.697 | 0.667 | 0.660 | 0.465 | 0.671 | 0.554 | 0.584 | 0.574 |
| 2b5i | 0.184 | 0.127 | 0.541 | 0.485 | 0.397 | 0.335 | 0.512 | 0.524 | 0.449 | 0.508 |
| 2gib | 0.536 | 0.695 | 0.529 | 0.658 | 0.675 | 0.316 | 0.919 | 0.541 | 0.496 | 0.830 |
| 1p4t | 0.923 | 0.777 | 0.921 | 0.955 | 0.700 | 0.486 | 0.860 | 0.860 | 0.887 | 0.950 |
| 1qgt | 0.805 | 0.535 | 0.709 | 0.646 | 0.815 | 0.507 | 0.715 | 0.742 | 0.805 | 0.757 |

Our model^1^ is constructed on the sequence dataset compiled from the bound structures; our model^2^ is constructed on the sequence dataset compiled from the unbound structures; our model^3^ is constructed on the BETOPE's dataset.
